# Adolescent and young adult mortality in Bangladesh: findings from household surveys

**DOI:** 10.7189/jogh.15.04193

**Published:** 2025-07-04

**Authors:** Ema Akter, Bibek Ahamed, Abu Bakkar Siddique, Pradip Chandra, Nasimul Ghani Usmani, Ridwana Maher Manna, Md Hafizur Rahman, Tasnu Ara, Md Shahidul Islam, Md Alamgir Hossain, SM Hasibul Islam, Mohammad Sohel Shomik, Anindita Saha, Md Akib Al-Zubayer, Abu Sayeed, Lubna Hossain, Toufiq Hassan Shawon, Shabnam Mostari, Qazi Sadeq-ur Rahman, Shafiqul Ameen, Sabrina Jabeen, Anisuddin Ahmed, Shams El Arifeen, Ahmed Ehsanur Rahman, Aniqa Tasnim Hossain

**Affiliations:** 1 Maternal and Child Health Division, International Centre for Diarrhoeal Disease Research, Bangladesh; 2 Nutrition and Clinical Services Division, International Centre for Diarrhoeal Disease Research, Dhaka, Bangladesh; 3 Management Information System, Directorate General of Health Services, Bangladesh; 4 Aspire to Innovate (a2i) Programme, Dhaka, Bangladesh

## Abstract

**Background:**

In 2021, over 1.5 million adolescents (aged 10–19 years) and young adults (aged 20–24 years) died globally, approximately 4500 deaths daily. However, research on causes and factors influencing deaths among adolescents and young adults in Bangladesh is limited. We aimed to address this gap by identifying the leading causes of mortality and the factors affecting adolescent and young adult deaths.

**Methods:**

We conducted two cross-sectional, population-based surveys in urban Dhaka (December 2022) and rural Sitakunda (May 2023), covering 53 680 households and 250 249 individuals, with 72 530 aged 10–24 years. Between 2018–22, 163 deaths in this age group were recorded. We conducted verbal autopsies using the World Health Organization tool and assigned causes of death using the InSilicoVA algorithm. With descriptive statistics, we reported cause-specific mortality and utilised Cox proportional hazards models to estimate associations with background characteristics.

**Results:**

Road traffic accidents were the primary cause of death, accounting for 10% (n = 17) among adolescents and 10% (n = 16) among young adults, followed by respiratory diseases (14% in adolescents and 3% in young adults). Older adolescents (adjusted hazard ratio (AHR) = 2.0; 95% confidence interval (CI) = 1.4–3.0) and young adults (AHR = 1.6; 95% CI = 1.1–1.4) had a higher risk of death compared to early adolescents. Individuals with high wealth status had a lower risk of death (AHR = 0.5; 95% CI = 0.3–0.8) compared to those with low wealth status. Adolescents and young adults with no education had a higher likelihood of dying compared to those with education (AHR = 5.6; 95% CI = 3.7–8.2).

**Conclusions:**

To prevent untimely mortality among adolescents and young adults, efforts should prioritise leading causes such as road traffic accidents and respiratory diseases, and address vulnerabilities among rural residents, the uneducated, and those with low socioeconomic status. We recommend strengthening existing health programs for adolescents and young adults to reduce preventable deaths.

Adolescents and young adults represent a significant portion of the population and are pivotal to the country's socio-economic development [1]. Their health and well-being directly influence the nation's productivity, growth, and future demographic trends. The global population comprises 24% of individuals between the ages 10–24, referred to as adolescents and young adults [2]. On average, 4500 people per day, or over 1.5 million people overall, died in 2021 among those in the age group of 10–24 years [3]. Rapid changes in body size, composition, and physiology occur during adolescence, the second most significant era of human growth and development [4]. Major shifts in health occur around puberty, as new health risks with potentially life-threatening consequences become prominent [5–7]. Reproductive maturity brings about risks for sexually transmitted diseases, including HIV, and for women, particularly in low- and middle-income countries, risks linked to pregnancy and childbirth [8]. Patterns of injury change with physical maturity, with young men in particular incurring trauma from war, violence, and traffic accidents. Puberty also indicates the onset of many mental health disorders of adulthood, which can be associated with a heightened risk of suicide [9]. Consequently, health profiles undergo rapid changes from early adolescence to young adulthood [10].

Despite representing a significant portion of the population and undergoing critical health transitions, adolescents and young adults have often been overlooked in global health and social policy, resulting in limited progress in improving their health outcomes [11]. This is particularly evident in Bangladesh, a country that has experienced commendable progress in improving under-five and neonatal mortality rates [12,13] but faces stagnation in the mortality rates among adolescents and young adults. Between 2000–23, Bangladesh reported the second lowest progress in reducing adolescent mortality rates in South Asia, a worrying trend that has persisted into the recent decades, with the adolescent (aged 10–19 years) death rate per 1000 decreasing from 9.49 to 7.42 between 2000–23 [13]. In 2020, more than 245 000 adolescents died in Bangladesh [13,14]. On the other hand, Bangladesh Burden of Disease Collaborators in the Global Burden of Disease 2019 report documented that, non-communicable diseases accounted for 14 of the top 20 causes of death, with stroke, ischaemic heart disease, and chronic obstructive pulmonary disease as the leading causes of death in the overall population [15]. The trend in mortality rate among adolescents and young adults in Bangladesh indicates a critical gap in the country's public health initiatives and underscores the need for a focused investigation into the causes and patterns of mortality in this age group.

Moreover, many low- and middle-income countries lack comprehensive vital registration systems capable of producing reliable and timely mortality data [16]. As a result, the existing estimates of youth mortality in these countries are based on modelling [16]. In Bangladesh, for instance, there is a paucity of data on the causes of death at the population level [17], with no national system for registering deaths and determining their causes [18]. While the Bangladesh Bureau of Statistics operates a Sample Vital Registration System that collects cause-of-death data through a lay reporting system, there are reservations about the accuracy of the cause-of-death information from this system [19,20]. To address this gap, researchers have turned to verbal autopsy methods to ascertain causes of death among children and adults, especially in settings where deaths occur outside a hospital. Recent studies have shown that verbal autopsy (VA) can yield cause of death information at the population level that aligns closely with that from high-quality hospital death certifications [21–23].

Research on the causes of deaths and risk factors associated with mortality among adolescents and young adults remains limited in Bangladesh. Therefore, we aimed to fill the knowledge gap by identifying and characterising the most common causes of mortality within this demographic in Bangladesh. We employed the VA method, utilising data from household surveys. By providing a detailed analysis of mortality patterns, underlying causes and associated factors, we seek to inform public health strategies and interventions tailored to the needs of Bangladesh's young population, ultimately contributing to the reduction of mortality rates and the improvement of health outcomes in this critical age group.

## METHODS

### Study design and sites

We conducted two cross-sectional, population-based household surveys. The first survey took place in urban Dhaka city in December 2022, followed by a second survey in the rural area of Sitakunda Upazila (sub-district) in May 2023 (Figure S1 in the **Online Supplementary Document**).

### Study population and sample size

The study population involved adolescents and young adults aged 10–24 years and the deceased individuals within the same age range who passed away between 2018–22.

In Dhaka city, we visited 26 487 households, of which 22 309 provided their consent to give an interview, encompassing 100 180 population (Figure S2 in the **Online Supplementary Document**). Within this group, we identified 28 677 individuals aged 10–24 years and recorded 61 deaths occurring between 2018–22. Similarly, in Sitakunda Upazila, our visits reached 37 606 households. Consent was obtained from 31 371 of these households, representing a population of 150 069. Among them, 43 853 were identified as being in the 10–24 years age group, and we found 102 deaths among those aged 10–24 years that happened between 2018–22. In total, we included 72 693 adolescents and young adults (28 738 from urban areas and 43 955 from rural areas), which far exceeds the minimum required sample size (Appendix S1 in the **Online Supplementary Document**).

### Data collection

We employed a structured tool for listing all regular members of the surveyed households and collecting basic demographic information for each individual. In addition, we recorded any instances of death occurring within the surveyed household between 2018–22.

We performed VA for any reported deaths that occurred between 2018–22We conducted a tab-based VA using an adapted tool from the World Health Organization (WHO) (2022) standard VA tool, version 1.2 (WHO, Geneva, Switzerland) [24].

### Data analysis

We used *R*, version 4.3.3 (R Core Team, Vienna, Austria) [25] and STATA, version 15 (StataCorp, College Station, Texas, USA) [26] to do the analysis. We considered several baseline characteristics: age in years (10–15, 16–19 and 20–24), sex (male, female), religion (Islam, others), place of residence (rural, urban), educational status (no education, educated) and wealth status (low, middle and high; derived using principal component analysis based on ownership of household goods).

We presented the descriptive statistics showing the alive status (deceased or alive) of individuals according to their background characteristics. We reported *P*-values from the χ^2^ test to assess the statistical significance of differences in death counts across groups. To determine the cause of death in deceased individuals, we utilised the *R* package ‘InSilicoVA.’ InSilicoVA [27] is a statistical algorithm that estimates the most likely joint probability distribution of cause-specific mortality fractions and probabilities of each cause for each individual death. InSilicoVA is available as open-source software (including source code) for the *R* statistical programming language. We reported the percentage distribution of causes of death among adolescents and young adults, as well as the cause-specific mortality rate per 100 000 individuals (adolescents and young adults) within this demographic. We also outlined the percentage distribution of place of death for each cause. We reported care-seeking behaviour using percentage distribution by background characteristics. We used χ^2^ tests to assess whether there were significant differences in cause-specific mortality rates across background characteristics, as well as in the distribution of place of death and care-seeking behaviour. To assess the survival probabilities related to background characteristics, we fitted a Cox proportional hazards regression model. We reported adjusted hazard ratio (AHR) along with its 95% confidence interval (CI) derived from the Cox proportional hazards model.

While conducting the survival analysis, a key to this process was the documentation of each individual’s status at the end of their observed period – referred to as event occurrence (for deaths) or censoring (for those still alive). Specifically, the occurrence of a death by the end of December in any year within our study period (2018–22) was classified as an event. Conversely, individuals who remained alive by the end of December in the corresponding year were categorised as censored in our analysis. To ensure the precision of the investigation, we created a separate cohort for each year within the timeframe of 2018–22. These cohorts included both the deceased and living individuals for each respective year, allowing us to track changes in the population’s health status over time.

## RESULTS

The number of deaths differed significantly across groups defined by background characteristics such as age, sex, place of residence, education, wealth status, and religion (**Table 1**). For example, 37% (n = 60) of the deceased were older adolescents (16–19 years), whereas this group made up only 28% (n = 20 242 individuals) of living population. Males represented a higher portion of the deceased at 56% (n = 91) compared to females at 44% (n = 72), while among the living, males constituted 47% (n = 34 141 individuals). The rural demographic accounted for 60% (n = 43 853 individuals) of the living individuals and 63% (n = 102) of the deceased. Individuals without education formed 4% (n = 3123 individuals) of the total sample, yet accounted for 21% (n = 34) of the deaths. Those from the low wealth status group contributed to 47% (n = 76) of the deceased.

**Table 1:** Distribution of adolescents and young adults by survival status (deceased or alive) according to background characteristics (showing the number of individuals alive in 2022 and recorded deaths between 2018–22*

Category	Alive	Death	*P*-value
Age in years			
*10–15*	26 923 (37)	42 (26)	0.043
*16–19*	20 242 (28)	60 (37)	
*20–24*	25 365 (35)	61 (37)	
Sex			
*Female*	38 385 (53)	72 (44)	0.035
*Male*	34 141 (47)	91 (56)	
Religion			
*Others religion*	7698 (11)	18 (11)	0.000
*Islam*	64 832 (89)	145 (89)	
Place of residence			
*Rural*	43 853 (60)	102 (63)	0.000
*Urban*	28 677 (40)	61 (37)	
Education			
*No education*	3123 (4)	34 (21)	0.000
*Educated*	69 407 (96)	129 (79)	
Wealth status			
*Low*	24 145 (33)	76 (47)	0.000
*Middle*	24 720 (34)	55 (34)	
*High*	23 633 (33)	32 (20)	

*Data are presented as n (%). *P*-values indicate differences in death counts across groups.

**Figure 1:**
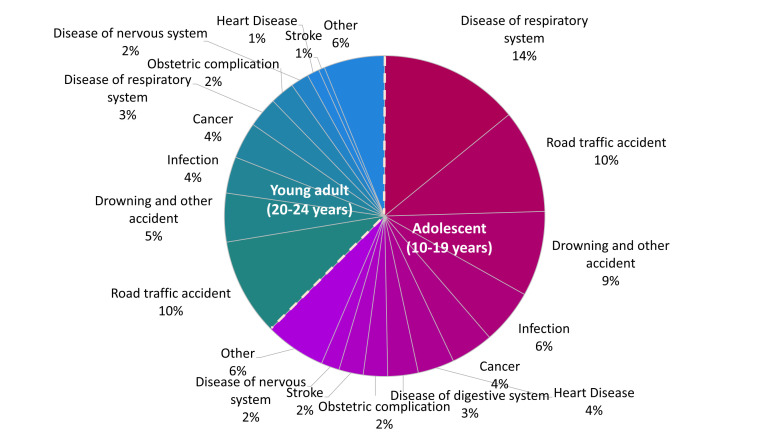
Distribution of cause of death among adolescents and young adults, presented in percentage distribution.

Road traffic accidents emerged as the leading cause of deaths, accounting for 10% (n = 17) among adolescents and 10% (n = 16) among young adults, followed by respiratory system diseases (14% (n = 23) in adolescents and 3% (n = 5) in young adults) (**Figure 1**). Other significant causes included drowning and other accidents such as accidental falls, assaults, and exposure to smoke, fire, or flames (9% (n = 14) in adolescents and 5% (n = 8) in young adults), infections (6% (n = 9) in adolescents and 4% (n = 6) in young adults), cancer (4% (n = 7) in both groups), and heart disease (4% (n = 6) in adolescents and 1% (n = 2) in young adults).

The mortality rate from road traffic accidents increased from 13 per 100 000 adolescents and young adults in 2018 to 15 per 100 000 adolescents and young adults in 2022 (Figure S3 in the **Online Supplementary Document**). Mortality attributed to disease of the respiratory system initially increased from four per 100 000 adolescents and young adults in 2018 to 11 per 100 000 adolescents and young adults in 2020, followed by a decline to six per 100 000 adolescents and young adults in 2022. Mortality rate from infection experienced a significant increase, from one per 100 000 adolescents and young adults in 2018 to 10 per 100 000 adolescents and young adults in 2022 (*P* = 0.004) (Table S1 in the **Online Supplementary Document**).

**Figure 2:**
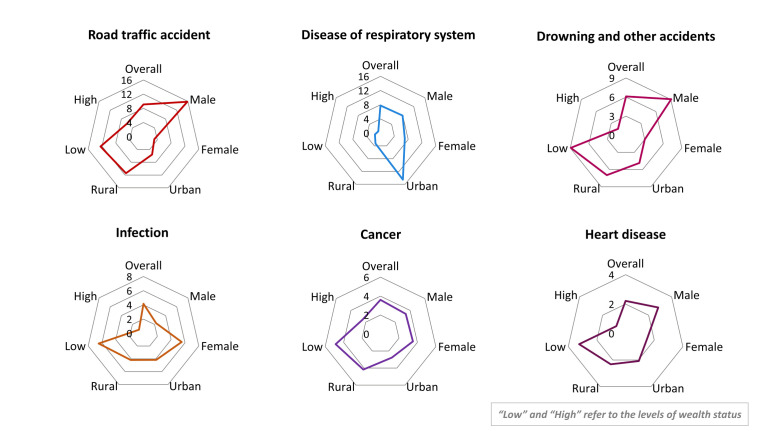
Cause-specific mortality rate (per 100 000 adolescents and young adults) by background characteristics.

Cause-specific mortality rates per 100 000 adolescents and young adults were highest for road traffic accidents (estimated deaths per 100 000 population per year = 9.1; 95% CI = 6.6–12.2), respiratory diseases (estimated deaths per 100 000 population per year = 7.7; 95% CI = 5.4–10.7), and drownings/accidents (estimated deaths per 100 000 population per year = 6.1; 95% CI = 4.0–8.9) (**Figure 2**). Road traffic accidents were significantly more common among males than females (*P* < 0.000) (Table S2 in the **Online Supplementary Document**), and drowning and other accidents were also more frequent among males (*P* = 0.016). Respiratory system diseases were significantly more common in urban areas compared to rural areas (*P* < 0.000). In terms of wealth, drowning and other accidents (*P* = 0.014) and infections (*P* = 0.021) occurred more often among individuals from lower wealth groups, with statistically significant differences.

Out of all deaths among adolescents and young adults, 38% (n = 62) of deaths occurred at home, 39% (n = 64) at hospitals or health facilities, and the remaining 23% (n = 37) in other locations (*P* = 0.038) (Figure S4, Table S3 in the **Online Supplementary Document**). For respiratory disease-related deaths, 39% (n = 11) occurred at home and 46% (n = 13) in hospitals, while 40% (n = 6) of infection-related deaths took place at home.

Around 58% (n = 95) of young adults sought medical attention, and the remaining 42% (n = 68) did not seek any form of care (Figure S5 in the **Online Supplementary Document**). Care-seeking varied significantly by background characteristics (Table S4 in the **Online Supplementary Document**). About 26% (n = 11) of early adolescents did not seek care before deaths (*P* < 0.000) (Table S4 in the **Online Supplementary Document**), in contrast to 48% (n = 29) of older adolescents did not seek care. Notably, one-third of females (n = 25) did not seek care (*P* < 0.000), while this figure stood at nearly half for males (n = 43). Urban individuals were more likely to seek care (62%, n = 38) (*P* = 0.007) compared to their rural counterparts (56%, n = 57). Care-seeking was also higher among individuals with high wealth status (72%, n = 23) (*P* = 0.001) compared to those with low wealth status (54%, n = 41).

**Figure 3:**
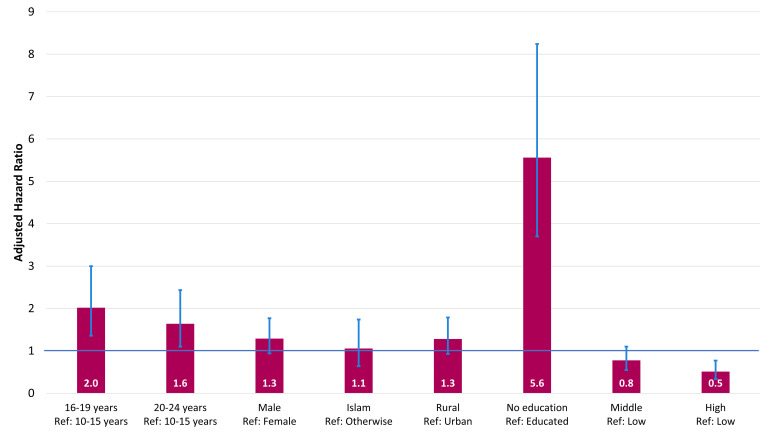
Factors associated with the survival of the adolescents and young adults (adjusted hazard ratio estimates from Cox proportional hazard model).

The findings revealed age, education and wealth status as significant determinants of the survival (**Figure 3**). Older adolescents (AHR = 2.0; 95% CI = 1.4–3.0) and young adults (AHR = 1.6; 95% CI = 1.1–2.4) had a higher risk of death compared to early adolescents. Adolescents and young adults with no education were at significantly higher risk of death (AHR = 5.6; 95% CI = 3.7–8.2) compared to those with education. Individuals with high wealth status (AHR = 0.5; 95% CI = 0.3–0.8) had a lower risk of death compared to those with low wealth status.

## DISCUSSION

In this study, we addressed a notable gap in data regarding the cause of deaths as well as the associated factors with survival among adolescents and young adults in Bangladesh. Road traffic accidents were the leading cause of death within this demographic, being responsible for 20% of the deaths, followed by diseases of the respiratory system, drowning and other accidents, including accidental falls, assault, exposure to smoke, fire and flame, infection, cancer, and heart disease. The findings revealed that older individuals, males, residing in rural areas, lacking education, and belonging to lower socioeconomic strata faced increased risks or hazards of mortality compared to younger individuals, females, those with urban living status, those educated, and those with higher socioeconomic status. Males comprised a higher proportion of the deceased at 56%, compared to females at 44%, while among the living, males constituted 47%. The rural demographic accounted for 60% of the living individuals and 63% of the deceased. Individuals without education formed 4% of the total sample, yet accounted for 21% of the deaths. Those from the low wealth status group contributed to 47% of the deceased.

In our study, road traffic accidents have been identified as a primary contributor to mortality among adolescents and young adults. A study conducted in Delhi, India also reported that the most active and productive age group, 15–35 years, was the most susceptible to fatalities resulting from road traffic accidents [28]. The reasons behind this trend among adolescents and young adults can be attributed to their increased mobility and exposure to road traffic, as they commute to work and school, unlike children who have less mobility and are often supervised by adults during road use [29]. Notably, there's a marked gender disparity in road traffic injuries, with males more frequently affected than females – a trend consistent with findings from Bangladesh and neighbouring countries [28–30]. This is probably due to the greater presence of men on the roads, engaged in work, business or educational activities, while women more often remain within the domestic sphere [29]. Our study findings highlighted that a lack of education and lower socioeconomic status are factors that increase vulnerability to road traffic-related deaths, a pattern also observed in a study from Ethiopia [31]. People from economically disadvantaged backgrounds often engage in work that requires extensive travel on hazardous roads, sometimes in vehicles that are overloaded or unsafe, increasing their risk of being involved in road accidents. There may be a deficiency in road safety education and awareness among people lacking formal education, belonging to lower economic groups, and residing in rural areas. This includes knowledge about safe driving practices, the importance of using seatbelts and helmets for protection. We found that rural areas are more prone to fatalities resulting from road accidents. A study from Bangladesh highlighted road safety issues, revealing through police-reported accident statistics that the incidence of accidents in rural areas, including on rural highways, is nearly twice as high as in urban [32]. This discrepancy can be attributed to poorer road infrastructure in rural areas, including inadequately maintained roads, poor lighting, a lack of pedestrian pathways, and insufficient road signs, which can increase the risk of accidents. To reach Sustainable Development Goal target 3.6: Halve the number of global deaths and injuries from road traffic accidents, a comprehensive approach to road safety is imperative [33]. This requires involvement from multiple sectors, such as transport, police, health, education, and actions that address the safety of roads, vehicles, and road users [33]. To reduce road traffic fatalities among adolescents and young adults, targeted interventions such as road safety education in schools and workplaces, stricter enforcement of helmet and seatbelt use, and improved access to safe public transportation are crucial. Additionally, awareness campaigns tailored to young drivers and pedestrians can help promote responsible road behaviour. During the COVID-19 pandemic, road traffic accidents declined due to lockdown measures and reduced mobility. However, as restrictions eased and traffic volume increased, fatalities surged again, highlighting the need for sustained road safety initiatives beyond temporary traffic reductions.

We found disease of the respiratory system as the second leading cause of death among adolescents and young adults, accounting for 17% of deaths in this group. Within this percentage, adolescents contributed to 14%, while young adults made up 3%. A review of population health data found that deaths due to lower respiratory tract infections were the most common cause among younger adolescents [10]. We observed variations in mortality from these diseases based on living environments, with urban dwellers found to be at a higher risk compared to their rural counterparts. This is aligned with the research suggesting that the risk of respiratory disease is higher in urban areas [34], likely due to increased exposure to air pollution from transportation, industry, and overall urbanisation, all of which adversely affect respiratory health [35]. The enhanced risk in urban settings can be attributed to the combined effects of air pollution and the spread of respiratory viruses. Efforts to reduce the incidence of acute respiratory infections among urban populations should therefore concentrate on reducing risk factors relating to overcrowding, socioeconomic disadvantage across the life course, as well as improving air quality [35].

Drowning and other accidents, including accidental fall, assault, exposure to smoke, fire and flame, emerged as a major contributor to mortality among adolescents and young adults. Not all adolescents have had the opportunity to learn swimming or water safety, which increases their risk around water bodies. Furthermore, communities characterised by high poverty levels and limited access to education can have higher rates of violence, affecting the safety of young individuals. Engaging in risky behaviours, such as playing with matches or lighters, without understanding the consequences, can lead to accidental fires. Our findings indicate that males are more susceptible to drowning and other accidents than females that is consistent with the findings reported by Rahman et al. revealed higher rates of both fatal and non-fatal drownings among males (fatal 19.0 per 100 000 per year and non-fatal 372.6 per 100 000 per six months) than females (fatal 12.8 per 100 000 per year and non-fatal 267.1 per 100 000 per six months) [36]. Additionally, accidents involving smoke, fire, and flames were more common among males [37], possibly due to risky behaviours, occupational hazards, or lack of safety measures. Drowning incidents mostly occur in rural settings [38], where the risk of drowning is found to be more than eight times higher than in urban areas [39]. This is partly because rural communities often lie in proximity to natural water bodies, such as rivers, lakes, and ponds, which are used for daily activities like washing, bathing, and fishing, thereby increasing drowning risks among unsupervised children and adolescents. The frequent exposure to water increases the risk of drowning, especially among children and adolescents who might be unsupervised. Implementing comprehensive educational programs that teach water safety, fire safety, and the dangers of risky behaviour is imperative to reduce these incidents.

Infection, including pregnancy-related sepsis, was observed as one of the major causes of adolescent and young adult deaths in our study. The WHO has highlighted infectious diseases, including maternal conditions (complications for pregnancy), as leading causes of death in this demographic [40]. Maternal conditions are becoming an increasingly important cause of death among young women in lower-income countries. This increase is due in part to limited access to health care services in these regions, which can be attributed to several factors, including inadequate infrastructure, a shortage of health care professionals, and financial constraints [40]. In Bangladesh, adolescent pregnancy remains a significant concern, with approximately 24% of women aged 15–19 years having begun childbearing [41]. We found that infection-related deaths are more prevalent among individuals from low wealth status. A study reported that an important adverse event experienced in childhood or adolescence, influenced by socioeconomic status, is poor health during these formative years [42]. Often, insufficient financial resources lead to inadequate hygiene, which contributes to recurring infections during this period [43].

Our findings reveal significant disparities in health care access and health care-seeking behaviour among adolescents and young adults, with mortality patterns varying by cause and place of death. A considerable proportion of deaths due to heart disease and cancer occurred at home, which may reflect barriers to accessing timely medical care. Financial constraints, limited awareness about early symptoms, and reliance on traditional or home-based care could contribute to this trend. Late-stage diagnosis, particularly for cancer, may also result in patients remaining at home rather than seeking hospital care, leading to poor survival outcomes. In contrast, infections and respiratory diseases accounted for a higher proportion of deaths that occurred in hospitals. This suggests that individuals with these conditions are more likely to seek care, but potentially at a later stage when treatment options are limited. Delayed health care-seeking behaviour, particularly among individuals from lower socioeconomic backgrounds, could lead to higher hospital mortality rates. Moreover, limited access to essential treatments, such as antibiotics and oxygen support, might contribute to these hospital deaths. Notably, a substantial proportion of deaths from drowning and accidents occurred during transit, highlighting potential gaps in pre-hospital emergency response systems. Timely access to emergency medical services is critical for reducing fatalities from injuries and accidents. Strengthening pre-hospital care, including ambulance services and first-aid training, could help improve survival rates for such cases.

Older individuals were found at a higher risk of death or lower survival likelihood in our study, aligning with WHO data showing that younger adolescents aged 10–14 years face the lowest mortality risk among adolescents and young adults [3,40]. This increase in risk is primarily due to external causes, such as accidents and falls [44]. In the context of Bangladesh, young adults aged 20–24 years, who typically have completed higher secondary education and are entering the job market, experience increased mobility compared to younger adolescents. This heightened mobility is associated with higher exposure to risks, contributing to a greater incidence of deaths among this age group. The study also found that young adults and older adolescents tend to seek less medical care during their last illness compared to early adolescents. This reduced health care engagement among older adolescents and young adults could contribute to their higher mortality risk. Older adolescents and young adults often prefer to manage health issues independently, either due to privacy concerns or a desire to take responsibility for their own care, unlike early adolescents, who generally rely on parents or guardians to make health care decisions.

The study also found that males are more vulnerable than females to deaths among adolescents and young adults, which is in line with the previous investigation wherein the risk rate for males was greater than twice as high as that for females [45]. This higher vulnerability among males may be attributed to their tendency towards more aggressive and riskier behaviours. Additionally, in Bangladesh, men are more frequently exposed to outdoor environments due to their roles in work, business, and education, which involve significant movement and exposure to road traffic. In contrast, females often have more restricted mobility, typically remaining at home and taking on household responsibilities [29]. Additionally, we revealed that the care-seeking status is lower among males compared to females. A study conducted in Lebanon among the students aged 17–21 years reported that women exhibited more formal behaviour towards health care than men [46]. Furthermore, women may have a higher level of health literacy than men in understanding medical forms [47]. This difference in health literacy and care-seeking behaviour contributes to the disparity in vulnerability to death between genders among young adults and adolescents.

The findings reveal significant disparities in health outcomes and care-seeking behaviours based on place of residence. Individuals residing in rural areas face higher risks of mortality compared to their urban counterparts, which aligns with a previous study that also reported considerably higher death rates among rural people compared to their urban counterparts [48]. This disparity may be partly attributed to external causes such as drowning and accidents, which are more common in rural settings due to occupational risks and inadequate safety measures. Additionally, urban residents were more likely to seek care than those in rural areas. Rural communities often face barriers, including limited health care infrastructure, long travel distances to facilities, and shortages of health care providers, all of which hinder timely and adequate care-seeking [49]. In contrast, urban areas benefit from a higher density of health care facilities, improved transportation systems, and greater health awareness, which collectively contribute to better health outcomes.

Our study revealed higher mortality rates among adolescents and young adults with no education or from lower wealth groups, consistent with previous findings [50]. A low level of education is often associated with poorer self-reported health, shorter life expectancy, and lower survival rates during illness [51]. Our findings also indicate that low-income and uneducated young individuals were less likely to seek medical care during their final illness compared to those from middle or high-income backgrounds. This disparity in health care-seeking behaviour plays a significant role in the increased mortality rates observed in low-income or uneducated young adults.

### Strengths and limitations

We conducted a rigorous analysis of causes of death among adolescents and young adults, incorporating samples from both rural and urban areas – which is limited in Bangladesh. While the study does not aim to be nationally representative, the inclusion of both settings provides valuable regional insights and enables more context-specific conclusions about mortality patterns. The study involves a substantial sample size (over 50 000 households surveyed). We utilised the standardised VA tool, a globally recognised method for determining causes of death in settings with limited medical certification. The majority of studies have recognised VA as a reliable and effective method for determining causes of death [22]. Additionally, the detailed breakdown of cause-specific mortality rates and their trends over time provides valuable insights for targeted public health interventions. We applied survival analysis techniques, allowing for a more robust examination of time-to-event data and providing insights into the risk factors associated with mortality among adolescents and young adults. Results regarding disparities in mortality based on socioeconomic status and education offer policymakers actionable insights for reducing health inequities.

Our study has several limitations. One limitation of this analysis is the relatively small frequency of deaths compared to the larger number of alive individuals, leading to a significant amount of censoring. However, the analysis remains important as it provides valuable insights into the associations between various characteristics (such as age, sex, education, and wealth status) and survival, while also offering useful information on the demographic factors influencing mortality in this population. Data collection took place during the COVID-19 pandemic, which posed significant challenges in accessing diverse populations and ensuring a representative sample. Despite employing robust quality control measures, the use of self-reported data to capture the number of deaths may have introduced some recall bias. However, it is worth noting that national health and demographic surveys typically use a five-year recall period for mortality reporting, whereas our study focused on more recent events within the past three years [52]. In addition, while the VA tool is a widely validated approach for collecting cause-specific mortality data in settings lacking medical death certification, its reliance on self-reported information may still limit accuracy. Furthermore, there is a potential for interviewer bias, as responses may be unintentionally influenced by the interviewer's tone, phrasing, or personal perspectives. Another limitation of the analysis is the absence of a physician review to confirm the cause of death. Without physician review, there is potential for misclassification or inaccuracies in determining the cause of death, which could affect the validity of the mortality data. However, recent estimates increasingly utilise the InSilicoVA method, a computer-based approach that uses machine learning algorithms to assess causes of death based on verbal autopsy data [53]. InSilicoVA method provides a valuable alternative to physician-based reviews, especially in settings with limited health care access.

## CONCLUSIONS

In this study, we provided critical insights into mortality patterns among adolescents and young adults in Bangladesh, a demographic vital to the country's socio-economic development. The findings reveal a concerning disparity in mortality outcomes, with higher death rates among males, older adolescents, rural residents, individuals with no education, and those from lower wealth strata. These results underscore the pressing need for targeted public health interventions and policy efforts to address the health disparities affecting this vulnerable group. Special attention must be given to causes of death, such as road traffic accidents and respiratory diseases. We recommend strengthening existing health programs for adolescents and young adults to reduce preventable deaths, with a focus on addressing these key health challenges.

## Additional material


Online Supplementary Document
