# Supplementary material for: Adolescent and young adult mortality in Bangladesh: findings from household surveys
Source: J Glob Health. 2025 Jul 4;15:04193. doi: 10.7189/jogh.15.04193 (PMC12231484; doi:10.7189/jogh.15.04193)
Supplement: Online Supplementary Document [file jogh-15-04193-s001.pdf]

Supplement to: Akter E, Ahamed B, Siddique AB, Chandra P, Usmani NG, Manna RM, Rahman MH, Ara T, Islam MS, Hossain MA, Islam SMH, Shomik MS, Saha A, Al-Zubayer MA, Sayeed A, Hossain L, Shawon TH, Mostari S, Rahman QS, Ameen S, Jabeen S, Ahmed A, Arifeen SE, Rahman AE, Hossain AT. Adolescent and young adult mortality in Bangladesh: findings from household surveys. J Glob Health. 2025;15:04193.

Figure S1: Study sites at Dhaka city and Sitakunda upazila.

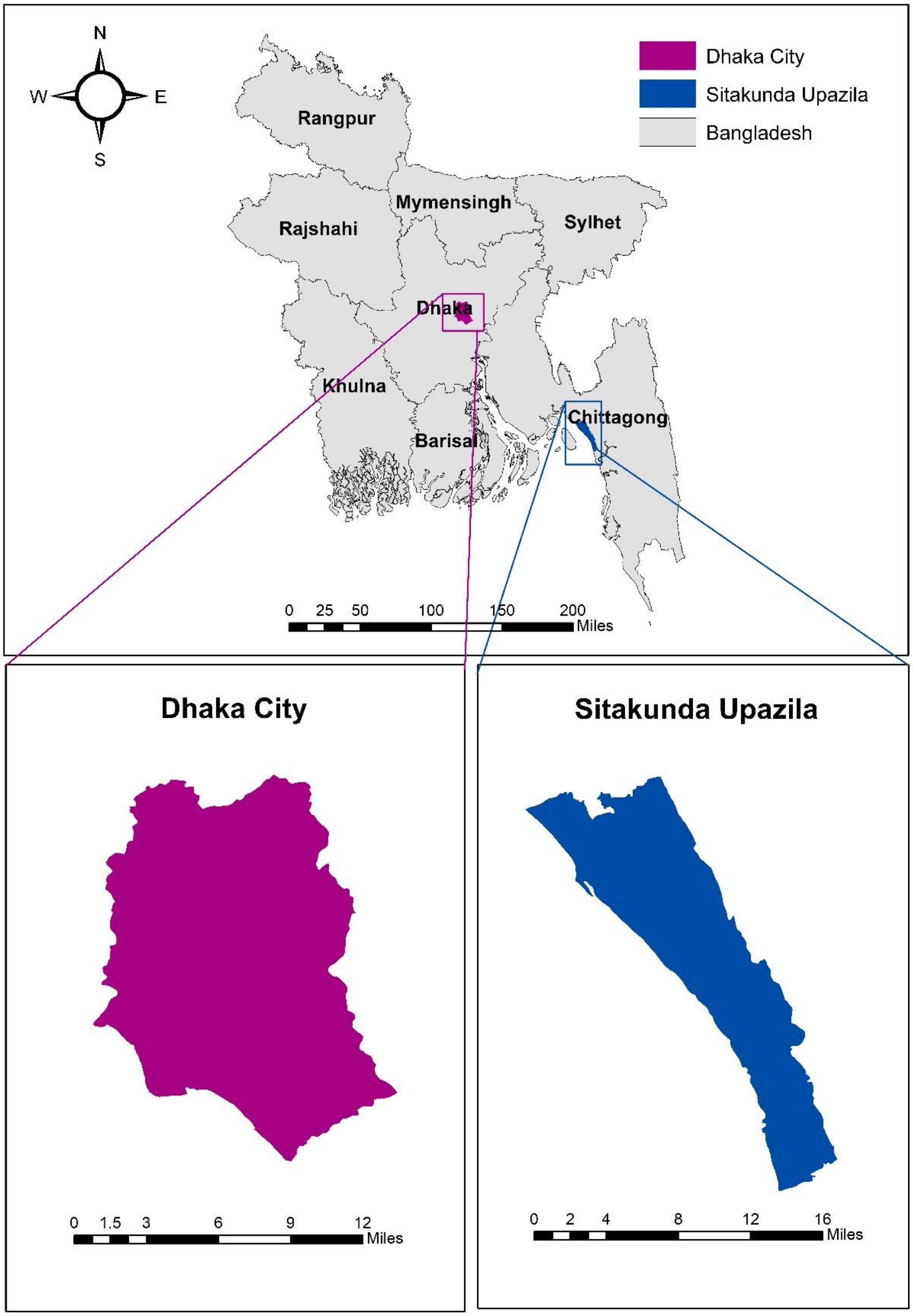

Figure S2: Flowchart of the study sample.

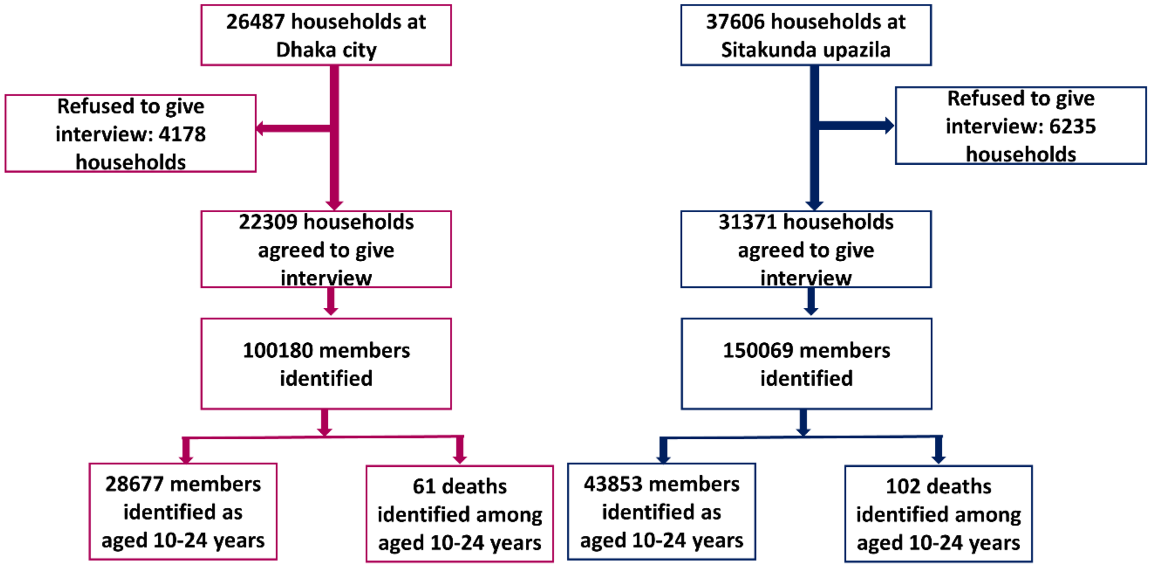

Figure S3: Trend in cause-specific mortality rate (per 100,000 adolescents and young adults) from 2018 to 2022.

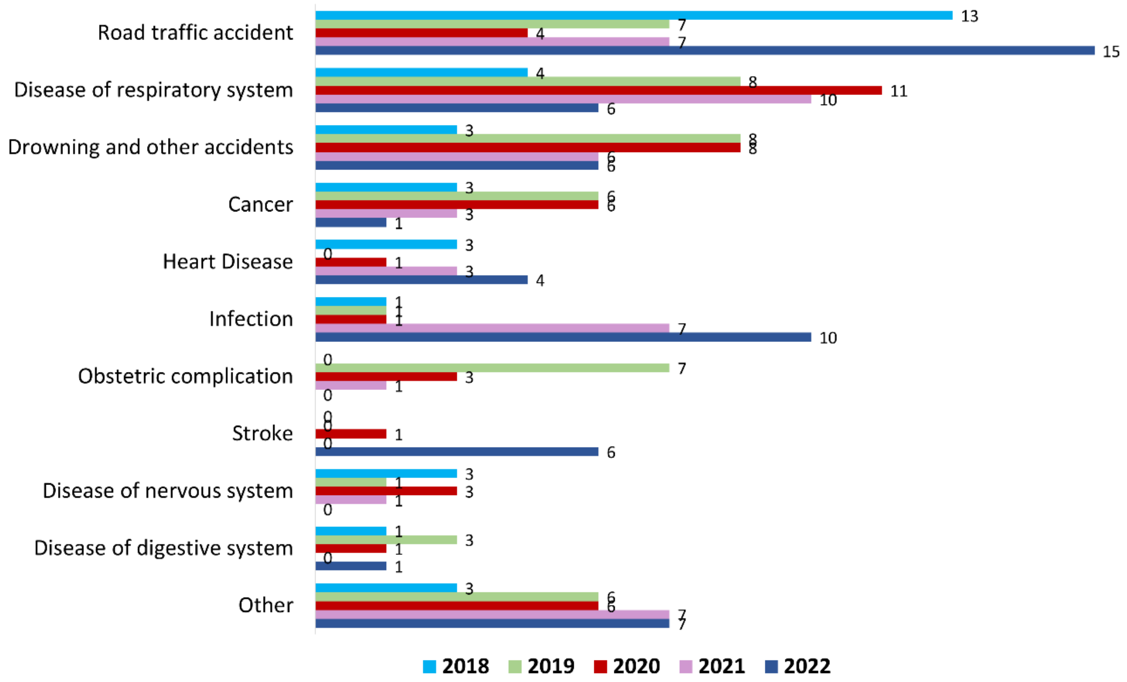

Figure S4: Place of death distribution by cause of death, presented in percentage distribution.

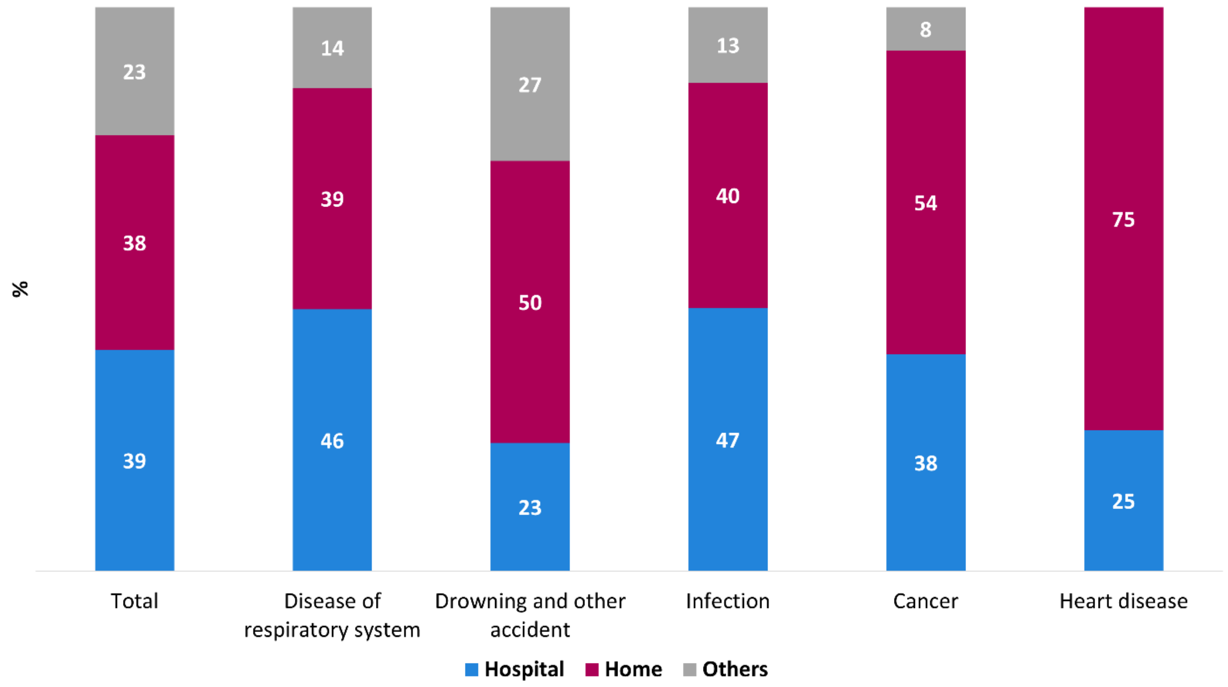

Figure S5: Care seeking practices by background characteristics, presented in percentage distribution.

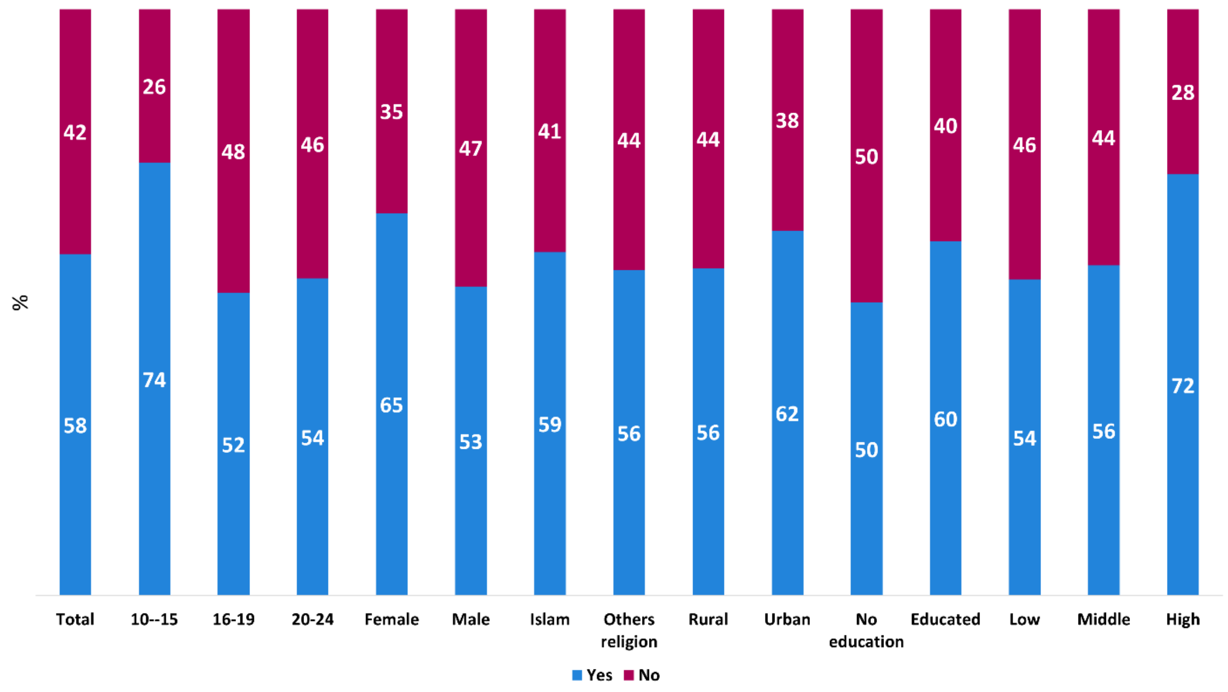

Table S1: Cause-specific mortality rate (per 100,000 adolescents and young adults) from 2018 to 2022

Causes	2018	2019	2020	2021	2022	P-value
Road traffic accident	13	7	4	7	15	0.622
Disease of respiratory system	4	8	11	10	6	0.689
Cancer	3	6	6	3	1	0.433
Disease of nervous system	3	1	3	1	0	0.248
Heart Disease	3	0	1	3	4	0.317
Other	3	6	6	7	7	0.268
Drowning and other accident	3	8	8	6	6	0.763
Disease of digestive system	1	3	1	0	1	0.527
Infection	1	1	1	7	10	0.004
Obstetric complication	0	7	3	1	0	0.317
Stroke	0	0	1	0	6	0.011

Table S2: Cause-specific mortality rate (per 100,000 adolescents and young adults) by background characteristics

Causes	Sex			Place of residence			Wealth		
	Male	Female	P-value	Urban	Rural	P-value	Low	High	P-value
Road traffic accident	16	3	0.000	6	11	0.072	16	19	0.098
Disease of respiratory system	8	7	0.757	15	3	0.000	2	1	0.056
Drowning and other accident	9	3	0.016	5	7	0.459	9	2	0.014
Infection	2	6	0.113	4	4	0.971	6	1	0.021
Cancer	3	4	0.947	3	4	0.518	5	2	0.333
Heart Disease	3	2	0.382	2	2	0.906	3	1	0.188

Table S3: Place of death distribution by cause of death, presented in percentage distribution

Causes	Hospital	Home	Others	P-value
Disease of respiratory system	46	39	14	0.145
Drowning and other accident	23	50	27	0.365
Infection	47	40	13	0.320
Cancer	38	54	8	0.172
Heart disease	25	75		0.075
Total	39	38	23	0.038

Table S4: Care seeking practices by background characteristics, presented in percentage distribution

Background characteristics	Care-seeking practices		
	Yes	No	P-value
10--15	74	26	0.000
16-19	52	48	0.715
20-24	54	46	0.365
Female	65	35	0.000
Male	53	47	0.459
Islam	59	41	0.003
Others religion	56	44	0.505
Rural	56	44	0.093
Urban	62	38	0.007
No education	50	50	1.000
Educated	60	40	0.001
Low	54	46	0.330
Middle	56	44	0.182
High	72	28	0.001
Total	58	42	0.003

Appendix S1: Sample size

While the primary surveys from which our data were drawn was originally designed to assess the impact of COVID-19 on mortality in urban area (Dhaka) and rural area (Sitakunda), we have retrospectively evaluated the adequacy of the sample size for our current analysis on adolescent and young adult mortality.

Using the most recent estimate of adolescent mortality in Bangladesh—7.42 per 1,000 population in 2023 (as reported by the World Health Organization's Global Health Observatory)—and assuming a 95% confidence level with a 0.005 margin of error, the minimum required sample size would be approximately 1,120 adolescents. This was calculated using the formula:

$$n = \frac{Z_{1-\alpha/2}^{2}\, p(1-p)}{d^2} = \frac{1.96^2 \ * 0.00742 * (1 - 0.00742)}{0.005^2} = 1120$$

In comparison, our study included a total of 72,693 adolescents (28,738 from urban areas and 43,955 from rural areas), which far exceeds the minimum required sample size. This large sample enhances the reliability and precision of our cause-specific mortality estimates in this population.
